# Biodynamers: applications of dynamic covalent chemistry in single-chain polymer nanoparticles

**DOI:** 10.1007/s13346-024-01665-z

**Published:** 2024-07-15

**Authors:** Lena Zeroug-Metz, Sangeun Lee

**Affiliations:** 1 Department of Pharmacy, Saarland University, Campus C 4.1, 66123 Saarbrücken, Germany; 2 Helmholtz Institute for Pharmaceutical Research Saarland (HIPS), Helmholtz Centre for Infection Research (HZI), Campus E 8.1, 66123 Saarbrücken, Germany

**Keywords:** Biodynamers​, Dynamic covalent bond​, Molecular dynamers​, Stimuli-responsive polymer, Polymeric nanoparticles​, Nanomedicine

## Abstract

Dynamic Covalent Chemistry (DCC) enables the development of responsive molecular systems through the integration of reversible bonds at the molecular level. These systems are thermodynamically stable and capable of undergoing various molecular assemblies and transformations, allowing them to adapt to changes in environmental conditions like temperature and pH. Introducing DCC into the field of polymer science has led to the design of Single-Chain Nanoparticles (SCNPs), which are formed by self-folding via intramolecular crosslinking mechanisms. Defined by their adaptability, SCNPs mimic biopolymers in size and functionality. Biodynamers, a subclass of SCNPs, are specifically designed for their stimuli-responsive and tunable, dynamic properties. Mimicking complex biological structures, their scope of application includes target-specific and pH-responsive drug delivery, enhanced cellular uptake and endosomal escape. In this manuscript, we discuss the integration of DCC for the design of SCNPs, focusing particularly on the characteristics of biodynamers and their biomedical and pharmaceutical applications. By underlining their potential, we highlight the factors driving the growing interest in SCNPs, providing an overview of recent developments and future perspectives in this research field.

**Graphical Abstract:**

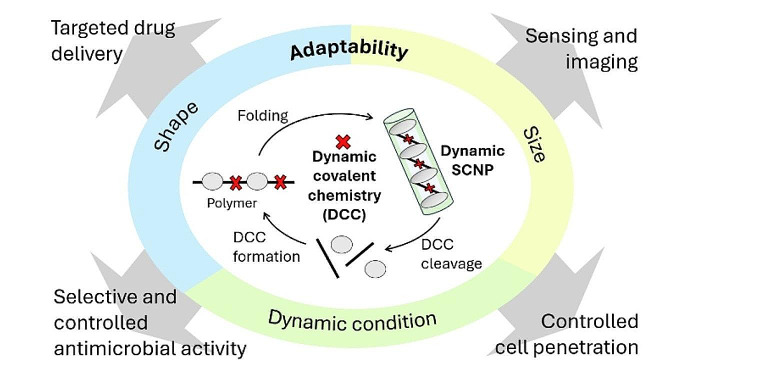

## Introduction

### ***Constitutional dynamic chemistry (CDC) and dynamic covalent chemistry (DCC)***

**Fig. 1:**
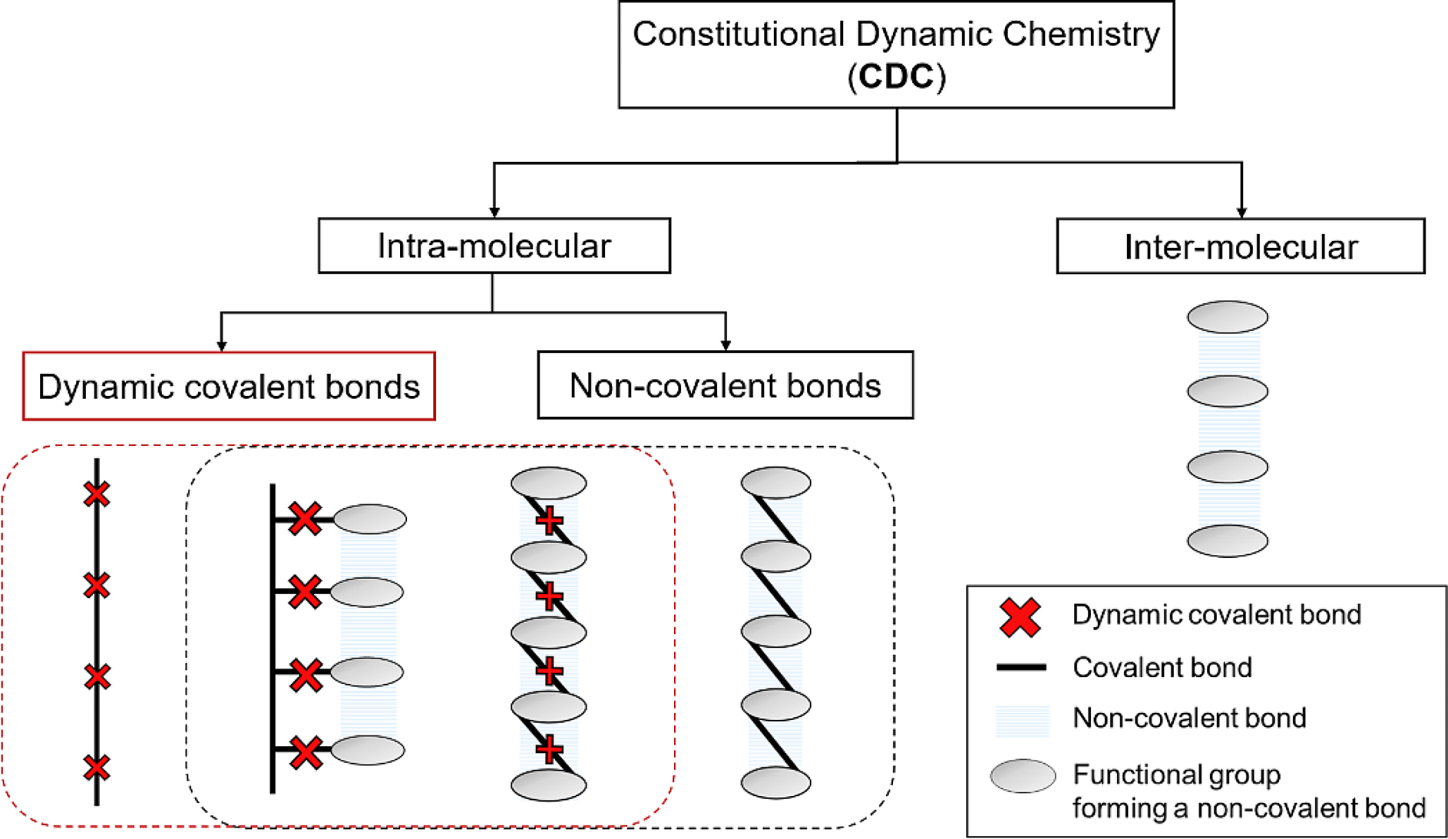
Overview and classification of constitutional dynamic chemistry (CDC) in polymers

Constitutional dynamic chemistry (CDC) involves generating dynamic libraries on supramolecular and molecular levels through the formation of reversible bonds among basic building blocks, as defined by Lehn [1]. The library’s composition, dictated by thermodynamic stability, aids in identifying stable structures and discovering self-assembling molecules [2–4]. External influences, such as changes in temperature or pH, can modify the library’s composition, making dynamic combinatorial libraries responsive tools for various applications, including discovering compounds with unique properties. Supramolecular chemistry includes the self-organization and formation of non-covalent bonds, found in. e.g., secondary, and tertiary structures in nature, vital for receptor generation and substrate-to receptor-recognition, only to name a few [5, 6]. By extending the dynamicity of supramolecular chemistry onto the molecular level, the field of dynamic covalent chemistry (DCC) is defined (see Fig. 1) [7]. Here, molecular components are linked by dynamic, reversible covalent bonds, forming all possible combinations of respective building blocks at thermodynamic equilibrium [3, 5, 7].

Utilizing reversible bond formation, DCC expands synthesis possibilities via the ability to adjust product distribution post-synthesis after the introduction of environmental stimuli, such as temperature, pressure, and pH [3, 5, 8]. The resulting formations can further be influenced by molecular targets, internal organization (self-recognition), or external factors (species binding), leading to the over-expression of selected products through target recognition-directed self-assembly [1, 9, 10].

### ***Dynamers***

In the field of polymer science, the incorporation of DCC via introducing reversible covalent bonds into the polymeric chain leads to the design of stimuli-responsive, tunable dynamic polymers (dynamers), opening up a variety of pharmaceutical applications such as drug delivery and imaging [3, 6, 8–11]. Inspired by the complex conformations observed in natural biopolymers, including secondary, tertiary, and quaternary structures, polymer scientists have become increasingly interested in designing synthetic polymers that self-assemble to create structures of such higher orders, mimicking substrate specificity and catalytic activity [12, 13]. This endeavor has led to the development of synthetic foldamers, which exhibit remarkable self-organization and self-assembly properties in secondary structures [2, 14, 15].

### ***Single-chain nanoparticles (SCNPs)***

Dynamers, derived from DCCs, have been developed into diverse biomaterials including injectable hydrogels, self-healing materials, and nanoparticles [12]. Moreover, by self-organizing into a secondary structure, dynamers are capable of forming single-chain nanoparticles (SCNPs), which is our particular focus in this manuscript. SCNPs are ultrasmall soft nano-objects formed by folding individual synthetic polymer chains through multiple intra-chain bonding interactions at high dilution [16–18]. This process, which involves intramolecular crosslinking interactions, results in the collapse of a single polymer chain and the formation of stable nanoparticles ranging from 1.5 to 30 nm in size, which resembles the sizerange of biopolymers or in general, proteins [19–23].

## Application of DCC in SCNPs

### Design basis of SCNPs incorporating DCC

In 2011, Murray and Fulton started to merge the concepts of DCC into the design of SCNP to create polymer-based assemblies with stimuli-responsive properties [24]. As described earlier, DCC utilizes reversible covalent bonds to form structures under thermodynamic control, allowing for modification of assembly constitution and functional properties [1, 3, 7]. By introducing dynamic covalent intramolecular bonds and crosslinking interactions into the single-linear polymer scaffolds, sub-20 nm nanoparticles are designed, capable of further structural reconfiguration in response to environmental changes or templates [25, 26]. This approach has offered the potential for error correction and refinement in binding sites, creating monodisperse, “intelligent” and adaptable nano-systems [5]. Focusing on dynamic covalent reaction-types, two principal mechanisms are included: First, exchange reactions, wherein one reactant displaces another to generate products with analogous bonding arrangements. Second, the formation of dynamic covalent bonds via condensation and addition reactions [5]. Covalent bonds that demonstrate dynamic characteristics include disulfides, acylhydrazones, imines, and boronic esters, contributing to the versatility of the resulting polymers [25, 27, 28].

One of the structurally adaptable SCNP-types is defined through the presence of dynamic covalent nitrogen-based bonds (imines and acylhydrazone) and organic, aromatic moieties such as carbazoles, which enhance the collapse of single-chain polymers into nanoparticles via π-π-stacking interactions [27, 29, 30]. As demonstrated by Giuseppone and Lehn, the advantageous characteristics of imine-based, covalent, and dynamic bonds are highlighted by their adaptability to changes in temperature and pH, laying the foundation for the development of stimuli-responsive materials [31].

With a focus on designing biocompatible, biodegradable, and optimally distributed nanomedicines in biological systems, polymer-based nanocarriers have caught special attention in recent years. In nanomedicine research, SCNPs are designed to improve drug and imaging agent delivery by targeting specific tissues. Mimicking protein folding, SCNPs are further explored for enzyme replacement, antibacterial and cancer treatment, making them a key focus in pharmaceutical research [19, 20, 27, 32]. Moreover, the reversibility of dynamic covalent chemistry (DCC), including redox- and pH-responsiveness, presents distinct advantages in biopharmaceutical applications [20, 26]. These features offer benefits in fields such as drug delivery and sensing, whether through the single chain nanoparticles (SCNPs) themselves or as part of a broader assembly of these nanostructures.

### Application of DCC in SCNPs

DCC involving disulfides and free thiols is well-documented, representing one of the earliest demonstrations of dynamic properties within this field. Dynamic disulfide crosslinking was demonstrated in SCNPs by employing a precursor polymer composed of hydroxyethyl methacrylate (HEMA) and pyridyl disulfide ethyl methacrylate (PDSEMA) [33]. Upon deprotection of the PDSEMA block, free thiols were generated, facilitating the formation of intramolecular crosslinks via disulfide bridges. Once formed, they can be cleaved under biological conditions through reduction, making them useful in stimuli-responsive drug delivery systems. Song et al. demonstrated the redox-responsive release of cargo from SCNPs by encapsulating Nile red as a model drug. The results indicated that the release of the loaded Nile red was significantly accelerated in the presence of a reducing agent (5 µM DTT).

**Fig. 2:**
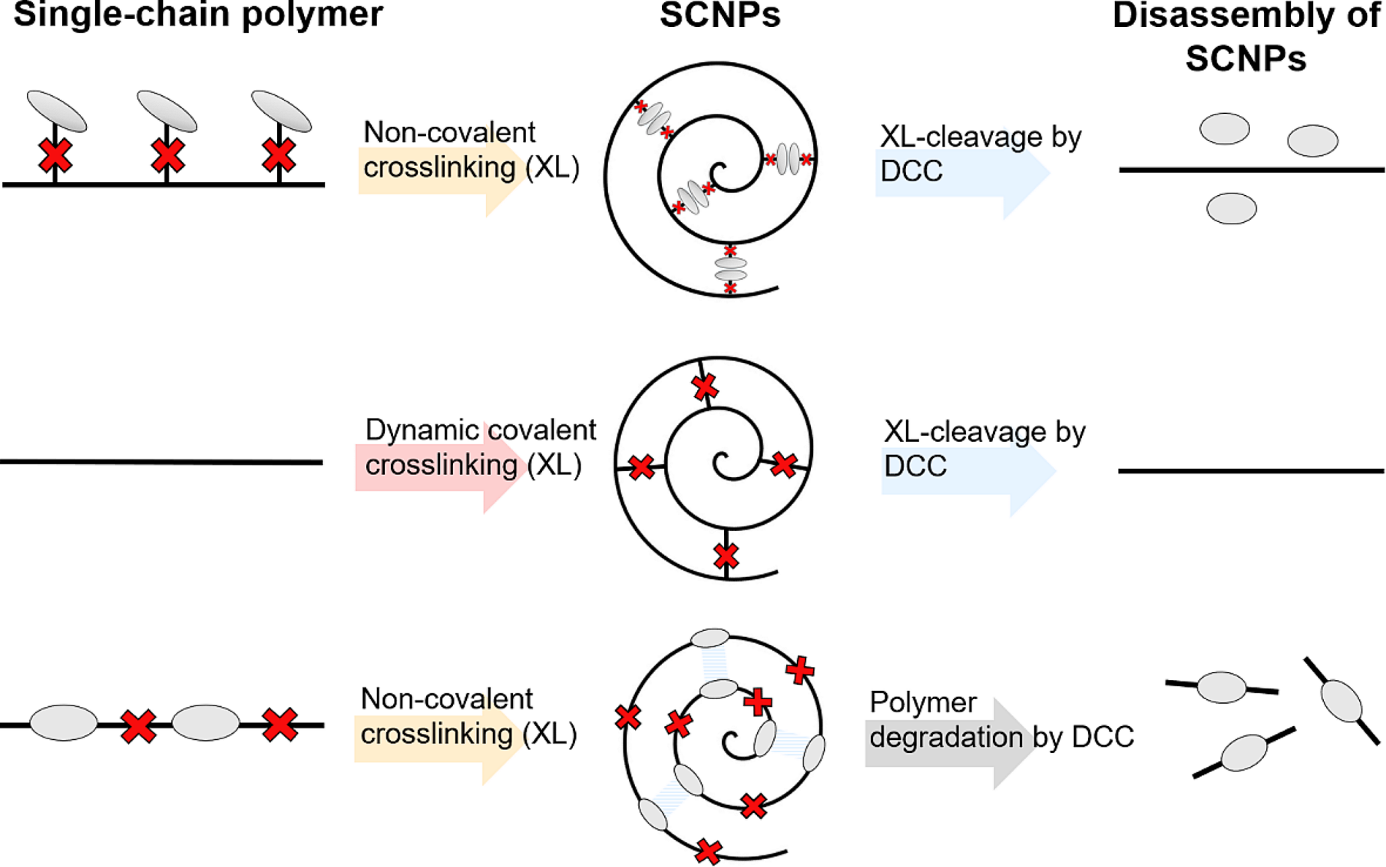
Overview of SCNP formation and reversibility with dynamic covalent chemistry. Legend of Fig. 1 explains the components in the figure

As mentioned earlier, acidity serves not only as a biomarker for tissues in diseases such as cancer and inflammation but also as a key characteristic of intracellular organelles such as endosomes and lysosomes. A polymer containing 2-(acetoacetoxy)ethyl methacrylate underwent functionalization through the introduction of a single enamine group, facilitating its interaction with ethylenediamine [34]. With the modified polymer, SCNPs were synthesized through crosslinking within the polymer by dynamic alkyl amine/alkyl diamine exchange. The pH-responsive cleavage of the DCC crosslinker and the dissociation of SCNPs were confirmed under acidic conditions, which can be further applied for disease targeting (**see** Fig. 2). In addition to forming stimuli-responsive SCNP through crosslinking using DCC within the polymer, DCC can also be incorporated into the backbone of the polymers, which subsequently leads to the formation of SCNP through self-folding [6, 8, 27, 29, 30, 35]. In our research on DCC-based SCNPs, we are investigating a polymer known as biodynamer.

## Biodynamers, a SCNP incorporating DCC

### Proteoid biodynamers

**Fig. 3:**
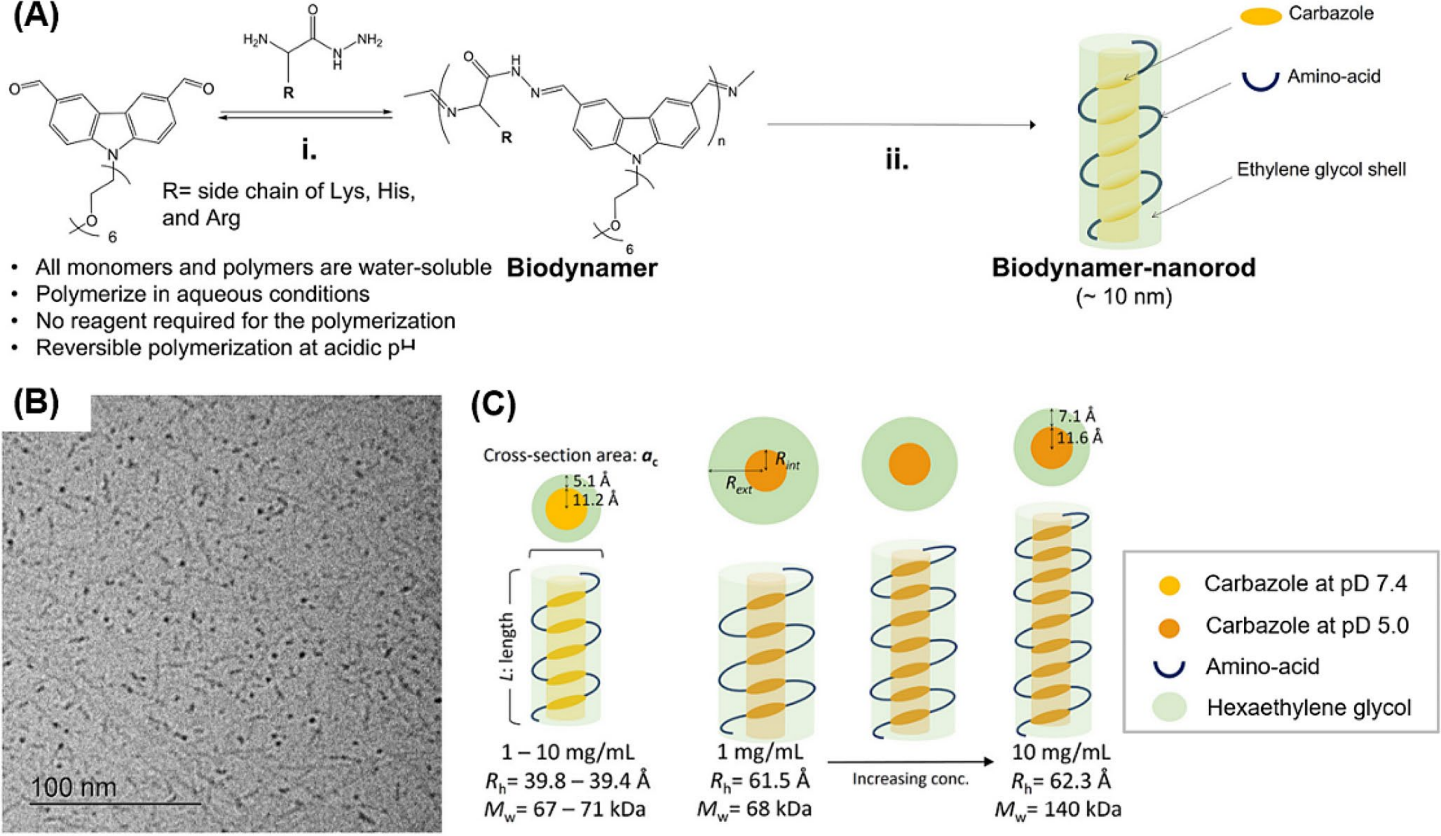
**(A)** Schematic illustration of proteoid biodynamers. After the DCC formation between aldehydes and amine/hydrazide (i), the biodynamers fold into a nanorod structure (ii). **(B)** cryogenic transmission electron microscopy (cryo-TEM) image of biodynamers showing their nanorod structure. **(C)** DCC-derived dynamic size and molecular weight changes of biodynamers under the acidic condition analyzed by small angle neutron scattering (SANS). Reproduced with permission [36]

Proteoid biodynamers, a novel category of SCNPs, are dynamic biopolymers characterized by their pH-responsive properties [27, 29]. As seen in Fig. 3A, the proteoid biodynamers are composed of two types of monomers, amino acid-hydrazides, and carbazole-dialdehydes conjugating hexaethylene glycol (CA-HG). Polymerized via DCC, reversible imine and acylhydrazone bonds are formed between the amines and aldehydes on the monomers through polycondensation in an acidic aqueous solution [6, 8, 29]. In particular, the imine bond formed in the polymerization of biodynamers exhibits high dynamicity, indicating significant reversible polymerization in mild acidic aqueous solutions.

The distinct feature of this biodynamer lies in the amphiphilic monomer, CA-HG. The CA-HG provides π-π stacking and hydrophobic interactions between repeating units, stabilizing the imines in the biodynamer backbone while keeping the dynamicity of the imines. Consequently, the formed biodynamer has relatively high molecular weights ranging from 70 to 130 kDa, despite the unstable imine bond being the main linkage in the formation of the biodynamer backbone. Also, they show dynamic behavior where their molecular weight decreases by 47.5% when the concentration is reduced tenfold from 10 to 1 mg/mL under acidic conditions (pH 5). Furthermore, a hydrophilic shell derived from HG contributes to the stabilization of the structure, facilitating the folding into nanorod-like nanoparticles, thereby indicating their nature as SCNPs (**see** Fig. 3B) [29]. Here, the HG-shell does not only drive and stabilizes the formation of biodynamers, but also improves their water compatibility and biocompatibility [29].Note that the biodynamers (Lys-biodynamers) exhibited a 44-fold higher IC50 value than their conventional poly amino acid form (poly-L-lysine). However, a degradation product of the biodynamers affected the cell viability when the biodynamers degraded completely into dialdehyde monomers with > 100 µg/mL.

The unique rod structure surrounded by HG-shell and biodegradability as well as compatibility make biodynamers well-suited for a range of biomedical applications, which are discussed later [36]. Nanorod structures can provide distinctive attributes in contrast to the prevalent spherical SCNPs (see Fig. 3C). In the case of biodynamers, characterized by a hydrophobic and π-π stacked core, they show significant interactions with drugs sharing similar characteristics to their core. Such interactions could be advantageous for drug loading and, therefore, drug delivery applications.

### Proteoid biodynamers in drug delivery

#### ***Peptide delivery***

Indeed, the biodynamers showed excellent drug loading of fluorescent dye-conjugated proteins (FITC-labeled albumin), with loading efficiencies of up to 78%, due to the interaction between the fluorophore of the drug and the core of the biodynamer [37]. In nanomedicine, the observed loading efficiency is exceptional as most systems suffer from low loading rates (1 ∼ 5 w%) [38–40]. Interestingly, the biodynamers assembled into a cylindrical nanostructure of a hydrodynamic size of 200 nm with the FITC-labeled albumin. At the same time, these features were not observed when removing the fluorophore from the cargo (albumin). These assembled SCNPs gradually degraded due to their dynamic nature (imines and acylhydrazones) under acidic conditions, allowing for the strategic release of loaded proteins. As demonstrated in the study, excellent loading and release control of proteins, along with biodynamer’s high potential as an mRNA vaccine to be discussed later, is expected to be useful for applications such as antigen delivery in vaccines.

#### ***Nucleotide acid delivery***

DCC-based SCNPs, in this manner, can be utilized for drug delivery by assembling into higher-order nanostructures. Particularly, the biodynamers can be advantageous in nucleotide delivery due to the dynamic changes in cell organelles like endosomes. By utilizing Arg-, His-, and Lys-hydrazide as monomers, we synthesized positively charged biodynamers [36]. Upon mixing with negatively charged mRNA, they formed dynamic particles (dynaplex) with a size of 120 nm. The dynaplexes efficiently delivered mRNA with toxicity levels 80 times lower than the most commonly used polymer, polyethylene imine (PEI), in similar applications. Additionally, they exhibited 1.4 times higher transfection efficiency in terms of transfected cell population compared to lipid-based vectors, the non-viral mRNA delivery vectors currently receiving significant attention. Such a high level of efficiency is attributed to the dynamic nature of biodynamers within the acidic endosomal environment, enabling effective endosomal escape. Notably, this capability is recognized as a crucial aspect in overcoming one of the key biological barriers to successful mRNA transfection.

#### ***Endosomal escape***

The effective escape of biodynamers from acidic conditions within endosomes can be interpreted in two possible ways, each originating from DCC and SCNP, respectively. Firstly, it could be due to the protonation buffering effect of DCC under acidic conditions. The buffering capacity of this polymer has been explored in previous studies, with particular emphasis on the high buffering capacity of amino acid hydrazide monomers released after degradation. Another possibility is that the structure of SCNPs, the amphiphilic rod structure of biodynamer, changes (shell size increase) and interacts with the membrane of the endosome under acidic conditions, thereby disrupting the endosome [36]. Many cell-penetrating peptides (CPPs), which were also explored as endosomal escape enhancers, have amphiphilicity and nanorod structures (α-helix structures) [41]. Moreover, it is suggested that secondary structural changes of the CPPs on the membrane represent an important mechanism in their functionality. In this regard, various CPP foldamers with well-defined secondary structures have been studied [42]. Therefore, further research on the structural impact of SCNPs in membrane interactions and disruption may broaden their design possibilities for biomedical applications. Indeed, it was observed that positively charged spherical SCNPs, which are anticipated to possess low buffering capacity due to 60 to 70% of quaternary amines, showed efficacy in cellular uptake due to their positive charges. However, unlike biodynamers, the spherical SCNPs remained confined within the endosome. Regarding the amphiphilicity of the biodynamers, their interaction with the cell membrane is further discussed in Sect. *Antimicrobial Agent*.

#### ***Cellular uptake***

 The size of SCNPs is advantageous for biomedical applications due to their suitability for effective cellular uptake. Bai et al. compared the cellular uptake efficiency of SCNPs with various sizes ranging from 7 to 40 nm [43]. The results showed that the smallest size, 7 nm, exhibited the most effective cellular uptake. Due to their smaller sizes compared to conventional polymer nanoparticles, SCNPs offer an advantage in cellular uptake. Typically, self-assembled, precipitated, and emulsified nanoparticles range from 30 to 250 nm for comparable applications [44–46]. Notably, it has been extensively studied that the molecular weight of the biodynamer and the resulting size of SCNPs depend significantly on concentration, composition, and structure. Under higher polymerization concentrations, larger nanorods are typically formed, with hydrodynamic sizes ranging from 7 to 11 nm, influenced by the concentration and acidity of the solutions [36]. Furthermore, as depicted in Table 1, the morphology, including size, is known to vary based on the side chain, specifically the amino acid monomer. Hence, incorporating DCC into SCNPs like biodynamers may offer an opportunity to control cellular uptake at specific targets, representing a potential avenue for future research for the design of DCC-based SCNPs.

**Table 1:** Proteoid biodynamers and their applications the values were obtained from [8]unless noted

Amino acid-Hyd	Mw	Morphology	Application	Remark
Arg	138,000	Nanorod Behavior	mRNA delivery [36] Peptide delivery [37] Adjuvants for antibiotics [47]	Endosomal escape Nanocomplex formation: human insulin, ovalbumin Emulsification: ovalbumin Potentiation effect of colistin (32-folds)
His	31,995	Globular Nano-Objects	Nucleotide delivery [36]	
Lys	71,738	Nanorod Behavior	mRNA delivery [36] siRNA delivery [48]	mCherry mRNA Survivin siRNA Endosomal escape
Lys-His-Arg	75,500 [36]	-	mRNA delivery [36]	mCherry mRNA Endosomal escape
Ser	21,447	Globular Nano-Objects	-	-
Thr	18,489	Globular Nano-Objects	-	-
Asp	6,277	Oligomer	-	-
Glu	3,000 67,300 [37]	Oligomer -	- Peptide delivery [37]	- Nanocomplex formation with GLP-1

### Potential application of biodynamers

#### ***Antimicrobial agent***

The advantageous characteristics of SCNPS regarding their interactions with the cellular membrane are not only due to their small size but also to their amphiphilic properties. Many SCNPs were formed by self-folding hydrophobic interactions in water [49–51]. Therefore, in addition to the previously discussed cellular uptake and endosomal membrane disruption, they can also be utilized as an antimicrobial material by disrupting bacterial cell membranes. The antimicrobial efficacy of SCNPs against Gram-negative bacteria *Pseudomonas aeruginosa (P. aeruginosa)* and *Escherichia coli (E. coli)* was assessed based on the functional groups present on the SCNPs [20, 52, 53]. Here, studies have shown that SCNPs with higher hydrophobicity demonstrate enhanced antimicrobial activity against pathogens by disrupting the cell membrane. This observation aligns with previous studies indicating that polymer nanoparticles with higher hydrophobic content tend to demonstrate greater antimicrobial efficacy [52, 53]. Considering these studies, it is expected that DCC-based SCNPs, such as biodynamers, will be effective in achieving increased targeting and reduced toxicity due to their efficient biodegradability. Therefore, we recently explored their application as safe, biodegradable, potentiators to enhance antibiotics. Consequently, due to the particular interaction with LPS on the Gram-negative bacteria (E. coli), we observed that the biodynamers formed pores on the bacterial membrane and enhanced the efficiency of the antibiotic up to 32-fold in the case of colistin [47].

#### ***Molecular sensors***

Finally, because DCC is responsive to external stimuli, including physical changes and the presence of chemicals in the body, SCNPs could potentially be used in sensing applications by integrating DCC into their design. Specifically, the inclusion of moieties that facilitate the easy readout of rapidly analyzable changes, such as fluorophores, is ideal. The development of fast and precise fluorescent molecular sensors is increasingly crucial in environmental and biological chemistry, especially since a disruption in essential metal ion homeostasis can quickly lead to the manifestation of various illnesses [54, 55]. Recent research on our proteoid biodynamers has primarily been directed toward drug and protein delivery, but their potential in ion recognition and sensing utilizing their distinct fluorescence emission remains largely unexplored [30, 36]. Derived from amino acids, biodynamers feature metal-ion-coordinating functional groups in their backbone, including acylhydrazones and imines, and offer the option to integrate ion-affine sidechains, such as histidine derivatives [56–58]. Given that the fluorescent core (CA) has been reported to potentially signal ion presence through changes in fluorescence, biodynamers are considered promising for detecting physiologically important metal ions such as Cu(II). This makes them suitable for applications that demand rapid and precise metal ion monitoring, which we are currently investigating [59–61].

## Summary and outlook

In this paper, we discussed the individual concepts of DCC and SCNPs, and explored their synergy in the biomedical field when combined. For SCNPs formed by factors such as amphiphilicity or π-stacking, they were able to load drugs with similar moieties effectively or form nanostructures of SCNP clusters. Based on this, the potential of SCNPs as drug delivery vehicles can be further explored from two perspectives: when drugs are loaded onto single-chain polymers with the small size of SCNPs (7 ∼ 40 nm), and when SCNPs assemble and form larger structures in the presence of drugs. In previous studies, it has been observed that SCNPs can regulate drug release more effectively at target sites when combined with DCC, suggesting promising future utilization strategies. Exploring the opportunities linked to the morphology of SCNPs, we further discussed the architectural design of SCNPs, which resembles protein secondary structures such as nanorods and is anticipated to broaden their functional scope to act like peptides. This includes similarities to CPPs or antimicrobial peptides (AMPs), as shown by foldamers. In this regard, biodynamers are potent candidates exploring this field as one example of DCC-comprising SCNPs, based on their unique self-folding forming nanorod structure. Although further investigation is necessary, the dynamic properties of biodynamers such as the DCC-originated dynamic rod-structure make them particularly effective for selective cellular uptake and targeted action within specific cellular organelles. Moreover, with their favorable functional groups and characteristics, biodynamers are promising candidates for ion recognition, potentially allowing for effective detection and analysis of ions across various applications. Last but not least, their amphiphilic nature significantly increases their water solubility, making them exceptionally biocompatible, dynamic polymer-based nanoparticles for a variety of pharmaceutical applications. Limitations, however, lie in the creation of synthetic polymers that include higher-ordered structures (tertiary, quaternary), mimicking folded biomolecules such as enzymes. Although further research is needed in the field of in-vivo applications and biodistribution, the potential of DCC-based SCNPs in pharmaceutical and biotechnological research is highly promising.

## Data Availability

Not applicable to this manuscript.

## Abbreviations

CDC: Constitutional dynamic chemistry
DCC: Dynamic covalent chemistry
CA: Carbazole dialdehyde
CA-HG: Carbazole dialdehyde conjugating hexaethylene glycol
Hyd: Amino acid-hydrazide
SCNPs: Single-chain nanoparticles
